# Surface Treatments with Dichloromethane to Eliminate Printing Lines on Polycarbonate Components Printed by Fused Deposition Modelling Technology

**DOI:** 10.3390/ma13122724

**Published:** 2020-06-15

**Authors:** Jorge Suárez-Macías, Juan María Terrones-Saeta, Francisco Javier Iglesias-Godino, Francisco Antonio Corpas-Iglesias

**Affiliations:** Department of Chemical, Environmental and Materials Engineering, Higher Polytechnic School of Linares, University of Jaen, Scientific and Technological Campus of Linares, 23700 Linares, Spain; terrones@ujaen.es (J.M.T.-S.); figodino@ujaen.es (F.J.I.-G.); facorpas@ujaen.es (F.A.C.-I.)

**Keywords:** 3D printing, additive manufacturing, fused deposition modeling, polycarbonate, surface treatments

## Abstract

Additive manufacturing, framed within the Industry 4.0. concept, is one of the processes that has witnessed greater development in the last years. Within this subject fused deposition modelling (FDM) printing technology is mainly dedicated to polymers and capable of providing components or elements of sufficient quality for different sectors. However, due to the process there can be a series of surface irregularities, which although they do not affect the required dimensional tolerances, they can cause problems in the useful life of the printed object in its interactions with the environment, as well as poor aesthetic qualities. Based on the above, this paper presents a series of chemical surface treatments capable of providing a surface that avoids undesired printing lines. For this purpose, fast, economical and environmentally sustainable treatments are used that obviously do not deteriorate the structure of the component or degrade the material surface. A complete study is therefore presented in which the different variables of the process are evaluated, as well as those of the printing technology, such as the layer height, coating, infill density, etc. The development of this project achieves a field of application of the detailed chemical treatment to obtain smooth surfaces, without degradation of the final part and with the appropriate dimensional tolerances.

## 1. Introduction

Additive manufacturing is one of the technologies that has been gaining momentum in recent years [1,2,3]. This fact is mainly motivated by the ease of production of parts, the lower cost in acquiring the different equipment as well as the diversity of materials available for the execution of the final products [4,5]. 3D printing, framed within this technology, provides products designed with very complicated geometries and extremely difficult to produce with traditional technologies [6,7]. So much so, that 3D printing is being used by different industries such as aeronautics, shipbuilding, railways and even the automotive industry to solve detailed problem, as well as to obtain prototypes or low production scale final parts [8,9,10,11].

It is therefore a new growing sector that is continuously innovating in equipment for its production, as well as in new materials and processes that maximize the quality of the final parts [12,13]. The quality demanded at a mechanical level in the mentioned sectors is maximum, and at the same time, a good aesthetic finish is also necessary to create an adequate image of the final product [14].

Within the different 3D printing technologies one is fused deposition modeling (FDM) [15]. This technology, developed in the eighties by Crump, uses thermoplastic materials of different nature to create the final components [16]. Its theoretical basis is simple, the equipment has an extruder that extrudes the filament of the desired thermoplastic at the right temperature to, through its movement in the X, Y and Z axes, deposit the molten material on a heated build plate and make the different sections of the final component [17]. The thermoplastic materials used are diverse, among them are ABS (Acrylonitrile Butadiene Styrene), PC (Polycarbonate), PLA (Polylactic Acid), PET (Polyethylene Terephthalate), PA (Polyamide), etc., and their applications are infinite thanks to their advantages such as: no post-curing, no excessive deformation, lower cost of equipment, capacity to combine different materials, etc. [18,19,20,21,22]. However, it has a series of disadvantages such as very slow speed or the need for exhaustive temperature control [16].

At the same time, the surface finish of the components manufactured by this technology displays a series of printing lines, inevitable for the product, due to its printing layer by layer technique, and which may not be aesthetically pleasing, or even serve to deposit dirt and/or organisms such that end up deteriorating the component [23].

To correct this aesthetic finish, paints have been used to smooth the surface or erosive surface treatments applied to eliminate the unwanted lines [24,25]. However, paints at the appropriate thicknesses can vary the dimensions of the component and can accumulate in complicated geometries [26]. It should be noted that the dimensional accuracy of FDM printing technology is enormous, being one of the variables to be taken care of and not to be despised. On the other hand, erosive treatments, normally with silica powder, are difficult to control in time, do not reach the whole surface when the geometry is complex and can mechanically deteriorate the part. 

Based on this, and in order to develop a fast and effective treatment that respects the dimensional variations of the product and obtains an adequate surface of the final product, the present work has been developed through the use of chemical surface treatments.

The chemical treatment of the product after its printing reaches the whole surface of the component, even if its geometry is complex, removes 3D printing lines and obtains a smooth and clean surface [27,28]. However, special care must be taken with the exposure time, as well as the manufacturing method, so that such treatment does not damage the component by being absorbed into it or vary the dimensions excessively after a prolonged exposure time. Therefore, it is essential to study the exposure time of components manufactured with different printing variables [29,30].

The material used in this study is polycarbonate, which has been and is used by all the plastic industries mainly due to its good mechanical behaviour and its different aesthetic properties and resistance in time. With this material several groups of samples were printed with different printing variables, mainly the layer height, the wall thickness and the infill density [31,32,33]. In turn, they were exposed to the widely used solvent methylene chloride (dichloromethane, DCM) for different times and then dried by an air current at room temperature. At the same time, dimensional variation and compressive strength tests were carried out to detect the effect of the treatment on the component.

The groups in which positive behavior was observed were subjected to scanning electron microscopy and Fourier transform infrared transmission spectroscopy (FTIR) to observe the microscopic quality of the surfaces and any deterioration of the polymer, respectively.

In this way, a series of printing conditions were obtained, as well as treatment times, in which the use of this chemical treatment was effective in removing the printing lines without damaging the part. In the following sections, the materials and methodology followed are detailed, as well as the results obtained from the tests of the different groups.

## 2. Materials and Methodology

### 2.1. Materials

The materials used in this paper are described below. Polycarbonate (PC) is the material for manufacturing the parts and dichloromethane the solvent used for the chemical treatment.

#### 2.1.1. Polycarbonate (PC)

The main material on which this paper has been based is polycarbonate. The choice of this material has been motivated by its extended use in diverse naval, aeronautical, railway and automotive industries, etc. and by its good mechanical characteristics and resistance in time, as well as by the lack of studies on the subject of chemical treatments that are within the scope of the present work.

Polycarbonate belongs to the group of thermoplastics. It is easy to work, mould and thermoform, being a polymer that presents functional groups united by carbonate groups in a long molecular chain. Polycarbonate offers a number of advantages over other polymers:

- Extremely high impact resistance.
- High transparency.
- High resistance and rigidity.
- High resistance to thermal deformation.
- High dimensional stability, i.e., high creep resistance.
- Good electrical insulation properties.
- High resistance to weathering, with protection against ultraviolet rays.

All these characteristics as well as its usual use in the plastic industry have motivated the choice of this material. The polycarbonate chosen for this work was a commercial material to facilitate its industrialization and make the results as widely applicable as possible. The properties of the polycarbonate used for the following research are shown in Table 1.

The properties reported in Table 1 are average of a typical group of samples. The 3D printing test samples were printed using a normal quality, 0.15 mm layer height 0.4 mm nozzle, 90% infill, 260 °C nozzle temperature, and 110 °C build plate temperature for electrical properties were measured on a 54-mm-diameter disk with 3 mm thickness printed in the XY plane, using the fine quality profile, 0.1 mm layer height a 0.4 mm print core, and 100% infill. The temperature of the laboratory during the printing phase and the execution of the tests is continuously regulated. The ambient temperature is 20 ± 1 °C and the humidity 45 ± 5%.

As mentioned above and can be corroborated in Table 1, polycarbonate has very good mechanical properties, among which a high tensile strength may be highlighted, in addition to having a very high glass transition temperature, which makes this material withstand high working temperatures.

These properties of the material, together with the advantages of additive manufacturing, make this combination a very interesting tool for the manufacture of components for different industries, especially those industries that require lower production volumes of the same component such as the space, naval and railway industries, as opposed to the large production of the same type of part in the automotive industry.

#### 2.1.2. Dichloromethane (DCM)

Dichloromethane is used as the main element for the surface treatment that eliminates printing lines. This product is used because it is a known polycarbonate solvent. Dichloromethane breaks the bonds of polycarbonate molecules and releases them for deposition. Therefore, it removes the printing lines and, if special care is not taken, dissolves the sample. At the molecular level, the smaller molecules of aichloromethane that are freely present release the larger polycarbonate molecules, increasing their entropy. That is to say, by carefully monitoring the exposure time of the piece surface, not only can the printing lines be eliminated, but it can create a smoother surface in which any pores that may have appeared during the printing are filled.

It is a colourless, volatile liquid with a characteristic odour. It reacts violently with metals such as aluminium, magnesium, sodium, potassium and lithium. It also attacks some types of plastics, rubber and coatings. This chemical is mainly used in the pharmaceutical industry and as a degreasing agent. Dichloromethane stabilized with 20 ppm of amylene has been used as a surface treatment in this work. Table 2 shows the characteristics of the dichloromethane used.

### 2.2. Methodology

As detailed above, the purpose of this work is the study of the appropriate printing conditions, according to the variables allowed to be modified for the manufacturing of the parts, and the exposure times to the chemical treatment for the elimination of the 3D printing lines. In this way, a smooth surface is obtained, without affecting the resistance of the final product.

The chemical treatment consists of immersing the part in methylene chloride for the stipulated time and then drying it with a current of air at room temperature. This treatment was carried out on all groups of parts with immersion times of 1, 2.5, 5, 7.5 and 10 s of exposure.

To execute the immersion of the components in the dichloromethane for the determined times, an automatic device was used in which the exact times were programmed and later they were submitted to drying.

The models for testing are composed of cubic bodies with 20 mm × 20 mm × 20 mm edge dimensions, printed with different variables. The printing variables used are those detailed below:

- *Layer height*. Layer height is one of the essential factors among the printing variables. As mentioned above, FDM technology is based on the successive printing of sections of the final component, these sections are therefore designed with a thickness corresponding to the thickness of the fused filament, it is therefore this thickness of fused filament that is deposited progressively that is the layer height. The thickness of the filament, or more correctly the layer height, has a significant influence on the surface finish, the correction of dimensions, the impression of details, etc., i.e., a quality impression will have a smaller layer height. However, production times increase exponentially the lower the layer height and, on average, the lower the material consumption. Professional equipment has very low layer height values, as the printed parts are perfectly dimensioned with respect to the design. These layer height values usually range for this professional equipment between 0.30 and 0.06 mm. Therefore, and in order to cover the entire range between layer heights, groups with three-layer heights of 0.06, 0.18 and 0.30 mm, respectively, have been developed in this project.
- *Infill density*. Most of the components 3D printed by FDM technology are not completely infilled inside but have a structure that supports the surface layer, as well as a wall thickness that will be detailed below. This is the case as long as no major mechanical characteristics of the designed part are affected. Therefore, and in order to base the study on all the possible cases, groups with 100% infill density were made, and others with increased wall thickness that will be detailed below and 20% infill density.
- *Wall thickness*. As mentioned, most components do not have 100% infill density on the inside, so to provide greater resistance to the surface layer there is this printing variable called wall thickness. The wall thickness varies in professional equipment from 0.6 mm to the complete filling of the components, so in this work we presented groups with wall thicknesses of 0.6, 1, 1.4 and 1.8 mm. Higher wall thicknesses would cause negligible surface variations in hardness so we proceeded to finish to 1.8 mm.

Based on these three print variables, the different groups of samples are presented in Table 3. Five groups of samples were made from each group of samples, corresponding to the five exposure times.

Of the fifteen groups of samples, several samples were made to subject half to detailed surface treatment. The remaining samples were not subjected to the surface treatment to evaluate the differences between the two.

Once the different groups of samples with the different exposure times were obtained, dimensional variation tests were carried out to determine the effect of the chemical treatment on the dimensions of the component; and a simple compressive strength test to determine the resistance of the component before and after the treatment. In this way it was possible to evaluate the quality of the surface treatment carried out according to all the variables of the process.

On the samples with suitable results, scanning electron microscope testd were carried out to corroborate the flatness of the surface, as well as the presence of any possible harmful micropores. The surface degradation of the polymer by chemical treatment was also evaluated using Fourier transform infrared transmission spectroscopy (FTIR). 

With this proposed methodology, clear and objective, it is possible to evaluate which printing variables involved in a surface treatment with dichloromethane that eliminates printing lines, creates a smooth surface of the piece and does not deteriorate its structure. Therefore, different combinations of printing variables and exposure times will be obtained that will allow the treatment to be developed successfully, and being, therefore, easily extrapolated to the industrial sector and managing to apply this treatment to a diversity of cases. At the same time, the proposed tests perfectly determine the viability of the treatment; on the one hand, the dimensional variation test limits the loss of geometry of the element; and on the other hand, the compressive strength test evaluates that the dichloromethane has not been introduced into the interior of the element and have not deteriorated the internal structure. With the results obtained, a graphic scheme will be obtained in which the various variables are represented and in which the combination of variables that successfully carry out a surface treatment and those that do not is identified. These various tests are described in the following subsections.

#### 2.2.1. Tests on the Different Groups of Samples

The main tests used to corroborate the suitability of the printing variables (layer height, wall thickness and infill density) for the different exposure times (1, 2.5, 5, 7.5 and 10 s) are the dimensional variation and simple compressive strength tests. These tests are described below.

##### Dimensional Variation

FDM printing technology has, among other notable advantages, the precision with which the parts are manufactured in terms of dimensions. It is therefore essential to respect this precision in the chemical treatment developed, trying to eliminate the 3D printing lines only and not affecting the most superficial layer.

In order to evaluate this characteristic, measurements were taken with an accuracy of 0.01 mm before and after the corresponding treatment. A measuring caliper with the specified accuracy was used for this purpose. Subsequently, those treatment times that do not make the printing lines disappear or those that, due to their longer exposure time, make the component lose part of its dimensions, were discarded. 

##### Compressive Strength Tests

As mentioned above, the chemical treatment developed is only superficial, so at all times it must be avoided that it penetrates into the interior of the component as it can collapse the internal structure when the infill density is not one hundred percent. The appropriate way to measure this parameter, and therefore discard those groups of samples that have suffered this type of damage, is through a compressive strength test. To this end, 15 more samples were made with the characteristics detailed in Table 3 to be tested in simple compressive strength tests without chemical treatment, comparing the values obtained with the subsequent tests on the groups of samples with the chemical treatment. In this way, it is possible to evaluate the effect of the chemical treatment on the internal structure of the sample and discard those groups of samples tested that may have been damaged by the surface treatment. Simple compressive strength as a measurable value is not the scope of this project, nor does it mean that it can be extrapolated to other cases, since it depends on various factors, including geometry. Therefore, the result of this test will be suitable, in the case in which the simple compressive strength before and after the treatment is similar, and null, if the simple compressive strength after the treatment is less than 5%.

The test of compressive strength was performed at a temperature of 20 ± 2 °C and with a speed of 5 mm/min until the detection of the breakage, decreased the load by a maximum of 30%. The test was carried out with a model AG-300kNX test press (Shimadzu, Kyoto, Japan).

#### 2.2.2. Surface Treatment Quality Tests

Through the tests mentioned in the previous section, the adequate exposure times for the different printing variables were evaluated, and therefore, a field of application of the chemical treatment was obtained that could be extrapolated to other components and different geometries. Once these characteristics were evaluated, the quality of the surface obtained was studied at a microscopic level, through the use of q scanning electron microscope, and the degradation of the polymer, by Fourier transform infrared transmission spectroscopy (FTIR).

##### Scanning Electron Microscope

With this equipment it was possible to observe the flatness of the surface, as well as the possible existence of microporosities, cracks or any other anomalous defect that could damage the viability of the component in the future. The scanning electron microscope used was a high resolution (FESEM), MERLIN (Carl Zeiss, Oberkochen, Germany) with EDX and WDX (Oxford Analytical, High Wycombe, UK) capabilities. It is an ultra-high resolution system that allows working with all types of samples both in image and analysis.

##### Fourier transform Infrared Spectroscopy (FTIR)

The FTIR Spectrometer used, is a Vertex 70 model (Bruker, Billerica, MA, USA), with all optical components required to work in near (NIR), medium (MIR) and far IR (FIR) regions. The test consists of the study of the spectra recorded before and after the chemical treatment, selecting those components that are subjected to the chemical treatment for longer without damaging them, and therefore, placing us in the most unfavourable case. The study of the spectroscopy before and after the treatment in an individualized way, as well as the superposition of both, entails the easy detection of peaks and therefore the evaluation of the similarity or difference in certain components of the same one.

## 3. Results and Discussions

The results of the tests mentioned in the methodology for the groups of samples mentioned in Table 3 and with exposure times of 1, 2.5, 5, 7.5 and 10 s are as follows.

### 3.1. Tests on the Different Groups of Samples

#### 3.1.1. Dimensional Variation

The dimensional variation before and after the treatment will assess the effect of the treatment on the final dimensions of the component. The results of this test for each layer height appear in the following subsections. It could be observed that even starting from different wall thicknesses the dimensional variations for each layer height were similar, something obvious if one thinks how the treatment affects the surface.

According to the data obtained for the dimensional variation, it can be seen that it is independent of the wall thickness as well as of the infill density, since similar values of dimensional variation have been obtained in all groups with the same layer height.

- Layer height of 0.06 mm

**Figure 1:**
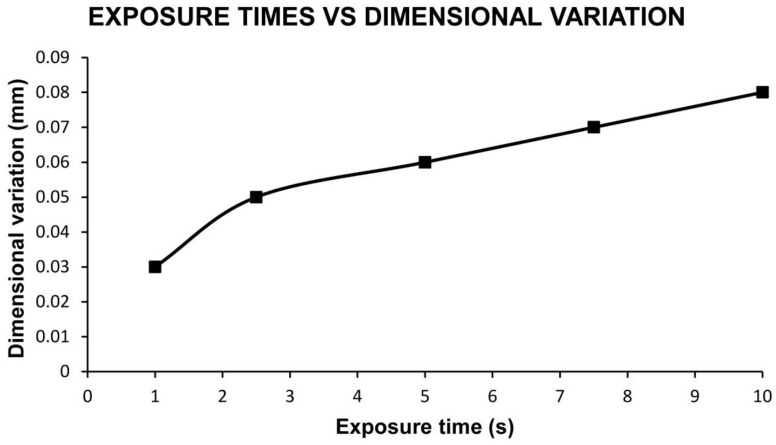
Dimensional variation for each exposure time and layer height of 0.06 mm.

The layer height results are shown in Table 4 and Figure 1.

It can be seen that with an exposure time of 2.5 s, a dimensional variation higher than the layer height is achieved and therefore the treatment capable of eliminating the 3D printing lines as could be observed on the corresponding components. Longer exposure times trigger a greater effect on the surface but without becoming alarming.

- Layer height of 0.18 mm

**Figure 2:**
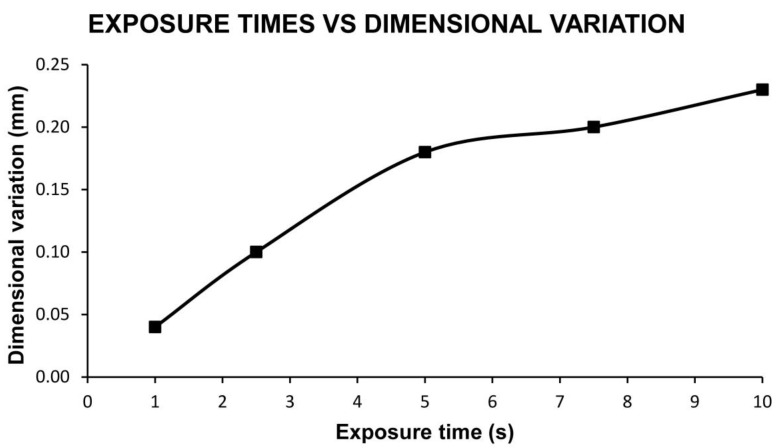
Dimensional variation for each exposure time and layer height of 0.18 mm.

The layer height results are shown in Table 5 and Figure 2.

Table 5 reflects how the increase in the exposure time of the components to the surface treatment, increases the dimensional variation. Taking into account that the dimensional variation is measured on one of the sides of the printed cube and that the layer height is 0.18 mm, can be establish that from exposure times of 5 s it is possible to remove the printing lines. Shorter times do not produce a flat component surface.

- Layer height of 0.30 mm

**Figure 3:**
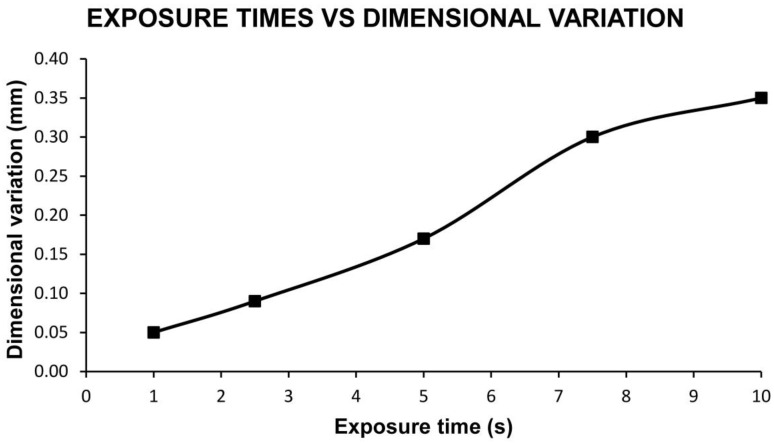
Dimensional variation for each exposure time and layer height of 0.30 mm.

The layer height results are shown in Table 6 and Figure 3. It can be seen that from the exposure time of 7.5 s onwards, a dimensional variation greater than the layer height is achieved and therefore this treatment capable of eliminating the 3D printing lines as could be observed on the corresponding components. This is due, as mentioned above, to the fact that the dimensional variation is measured on the side of the manufactured cube (component). Therefore, dimensional variations greater than 0.30 mm eliminate printing lines that have dimensions related to the layer height used.

The results of the dimensional variation tests for the three large groups, as a function of layer height, reflect how not all treatment exposure times are suitable for eliminating printing lines. Based on the operation of printing by FDM technology, the printing lines will have a height equal to half the layer height. Since the dimensional variation is measured on the side of the tested sample, a dimensional variation at least equal to the layer height is necessary to eliminate the printing lines.

On the other hand, a greater effect of the surface treatment is easily observed in the components with higher layer height. This is due to the lower quality they possess and the greater ease with which dichloromethane can penetrate them and cause greater dimensional variations.

In short, this section presents a series of results based on the ability to eliminate the printing lines. On components with a layer height of 0.6 mm an exposure time of more than 2.5 s is required, on components with a layer height of 0.18 mm an exposure time of at least 5 s is required and on components with a layer height of 0.30 mm an exposure time of more than 7.5 s is required. However, these results refer only to the elimination of the printing lines, so it must be taken into account that it does not affect the structure of the component. This issue is addressed by the compressive strength test, which evaluates the effect on the internal structure of the component and further limits the possible variables for successful treatment. 

#### 3.1.2. Simple Compressive Strength

The compressive strength test is performed for the exclusive purpose of evaluating the affection of the surface treatment on the structure of the component. The surface treatment must not affect the internal structure. Therefore, if the compressive strength of the components decreases after the chemical treatment, the results will be null. In other words, they would be null for the detailed printing variables and the exposure times to the dichloromethane treatment.

On the other hand, if the compressive strength is the same before and after the treatment, the surface treatment is considered adequate, as it does not affect the internal structure. The results are presented as acceptable or rejectable, since the study of the compressive strength and its variations is negligible. This fact is due to the fact that the compressive strength depends on the printing variables and the geometry of the component, so it is not extrapolatable to the generality. The results of this test are shown below in the following sections as a function of the layer height.

- Layer height of 0.06 mm

The values of simple compressive strength with a layer height of 0.06 mm and according to different wall thicknesses and exposure times are shown in Table 7.

From the data in Table 7, it is deduced that given the small thickness of the layer height, the treatment during the exposure time does not penetrate into the interior of the component and does not affect its mechanical characteristics. Except for wall thicknesses of 0.6 mm and exposure times greater than 7.5 s, as well as wall thicknesses of 1 mm and exposure times greater than 10 s.

- Layer height of 0.18 mm

The values of simple compressive strength with a layer height of 0.18 mm and according to different wall thicknesses and exposure times are shown in Table 8.

Table 8 reflects that a layer height of 0.18 mm behaves similarly to a layer height of 0.06 mm, as the compressive strength is acceptable in most cases. However, for wall thicknesses of 0.6, 1 and 1.4 mm, and exposure times of more than 5, 7.5 and 10 s, respectively, the treatment penetrates the component and damages its resistance. It is therefore an intermediate case in which the importance of the layer height in the 3D printing is denoted.

- Layer height of 0.30 mm

The values of simple compressive strength with a layer height of 0.30 mm and according to different wall thicknesses and exposure times are shown in Table 9.

In this case, it can be seen that a greater layer height has a negative influence on the correct performance of the surface treatment. This is due to the fact that dichloromethane penetrates the internal structure through the different pores, dissolving it and decreasing the compressive strength. However, a greater layer height is corrected by a greater wall thickness and shorter exposure times, as shown in the data, as reflected in Table 9.

In short, and with the results of both tests, taking as a premise the dimensional variation such that it eliminates the printing lines and that the simple compression resistance is adequate, Table 10 summarizes the groups that can be successfully treated by this surface chemical treatment.

Based on these results these are groups of detailed impression variables and exposure times in which the chemical treatment can be successfully performed. This data is therefore a quick guide to ensure that the surface chemical treatment performs well.

**Figure 4:**
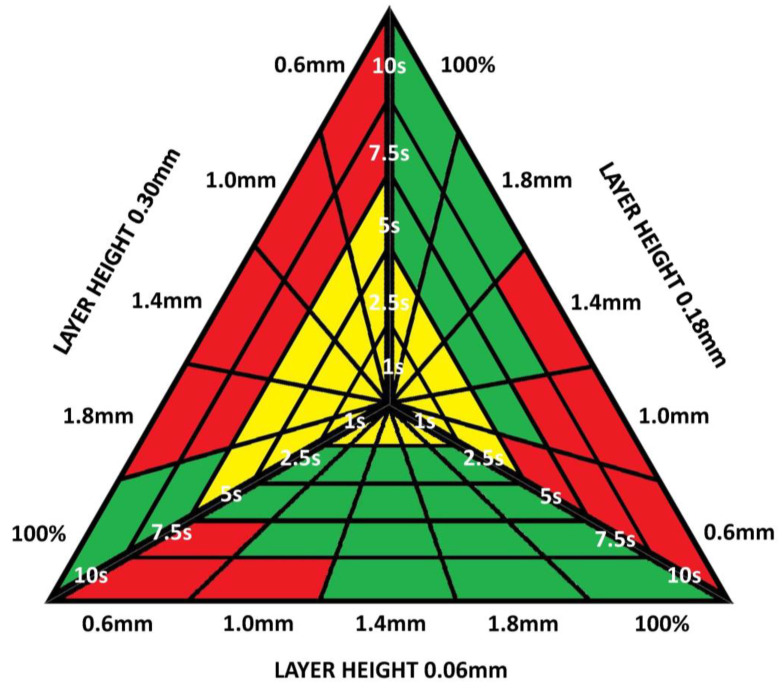
Graph of combination of printing variables and exposure times to dichloromethane that produce a correct surface treatment. In green, combinations of acceptable variables; in red, combinations of rejected variables; and in yellow, combinations of variables that do not eliminate the impression lines.

Figure 4 shows in a graph the groups of samples that are acceptable for treatment with dichloromethane, according to the printing variables and the times of exposure to the treatment. The acceptable results are shown in green, in red the combination of variables that deteriorate the piece and in yellow the variables that do not deteriorate the piece, but do not eliminate the printing lines.

### 3.2. Quality Testing of Surface Treatment

Once the appropriate groups of samples and exposure times have been evaluated, we proceed to perform scanning electronic microscopy and the Fourier transform infrared transmission spectroscopy (FTIR) quality tests to evaluate the quality of the treatment.

#### 3.2.1. Scanning Electronic Microscopy

For the scanning electronic microscopy test, different samples were taken from the groups of samples in which the surface treatment had been favourable with different exposure times, as well as components without chemical treatment. These samples were metallized with carbon for the microscopy study.

The surface of all the components was observed with acceptable results, determined by the compressive strength and dimensional variation tests, and the existence of discontinuities that could suppose detrimental to the part was evaluated. This section shows the images obtained with the scanning electronic microscope for the printed polycarbonate pieces with a layer height of 0.30 mm, 100% infill density and exposure times of 10 s. Although the images of all surfaces of the accepted parts were similar, this group was selected for different reasons. On the one hand, these are the samples that greater effect produces the treatment, as can have been observed in the compressive strength tests. And on the other hand, they are the parts that the treatment should eliminate the highest height of the printing lines, since the layer height is 0.30 mm.

**Figure 5:**
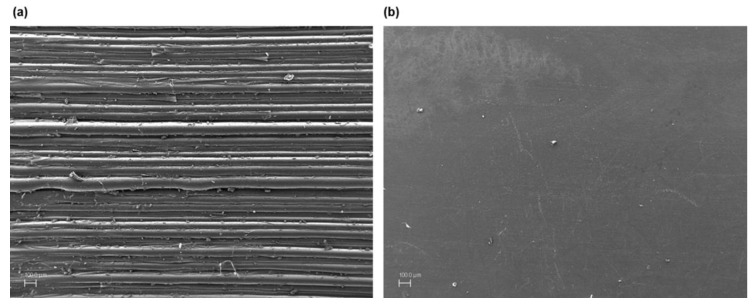
Polycarbonate component manufactured with 3D FDM printing without chemical treatment 250× (**a**) and with dichloromethane chemical treatment 250× (**b**).

**Figure 6:**
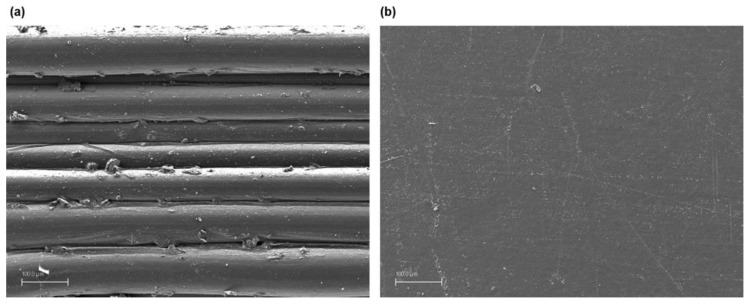
Polycarbonate component manufactured with 3D FDM printing without chemical treatment 1000× (**a**) and with dichloromethane chemical treatment 1000× (**b**).

**Figure 7:**
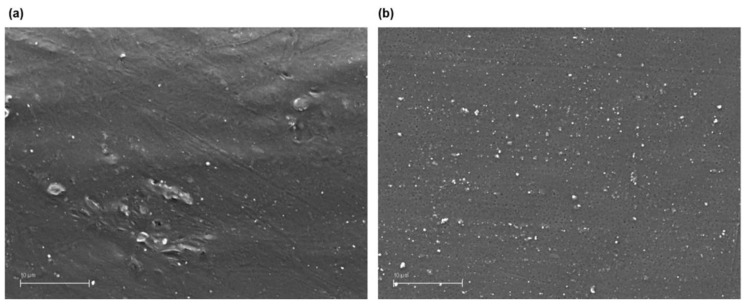
Polycarbonate component manufactured with 3D FDM printing without chemical treatment 15,000× (a) and with dichloromethane chemical treatment 15,000× (**b**).

The following Figure 5, Figure 6 and Figure 7 detail the surface image for the sample group commented on before and after the treatment, as well as with different amplifications.

The figures show a clear difference between chemically treated and untreated components, reflecting a completely smooth surface with no deterioration of the material. Figure 7 with the highest magnification, 15,000×, displays deeper and more extensive porosities in the components without chemical treatment than those with chemical treatment, with no defects or discontinuities in the latter. On a microscopic level the treatment behaviour is excellent compared to the initial components and to other types of much more aggressive surface treatments.

#### 3.2.2. Fourier Transform Infrared Spectroscopy (FTIR)

**Figure 8:**
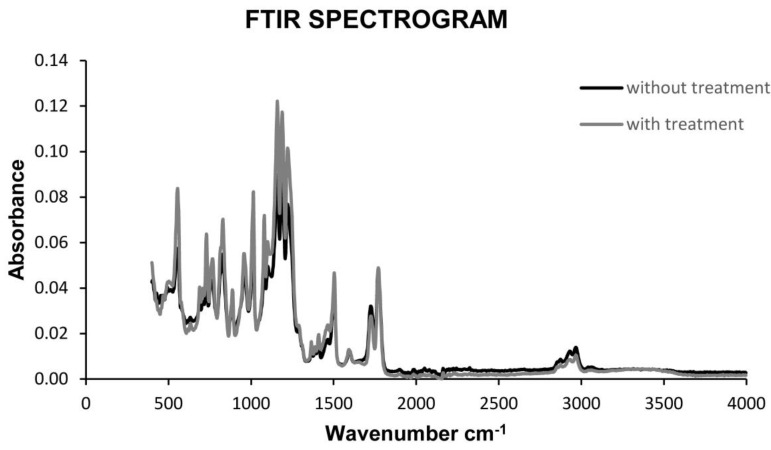
Comparison FTIR spectrogram for polycarbonate components manufactured with 3D FDM printing with and without chemical treatment.

This test was carried out for components without chemical treatment and with chemical treatment in the longest exposure time, 10 s, in order to study and compare the results with the most unfavourable case. The results obtained for untreated components were similar to those obtained for chemically treated components, regardless of the group to which they belonged. The spectroscopy results for parts with and without chemical surface treatment are shown in Figure 8.

The comparison of both spectrograms clearly reflects the non-incidence of the treatment on the degradation of the polymer. The peaks presented are in full agreement and their positions have not changed, therefore it can be concluded that there is no significant degradation. On the other hand, the increase in the intensity in the spectrogram of the parts with surface treatments is due to a better positioning of the surface during the test. Therefore, it is not an influential characteristic in the evaluation of the treatment’s viability. In short, it can be concluded that the surface treatment has generated a smooth surface, without discontinuities, and that the polymer after the treatment has not experienced any degradation that would makes the part unusable.

## 4. Conclusions

The analysis of the different tests reflects a series of partial conclusions that converge in the final conclusion. The final conclusion is to confirm the suitability of carrying out chemical surface treatments with dichloromethane on polycarbonate parts manufactured by FDM printing technologies. The partial conclusions of the study are explained below.

- The chemical surface treatment with dichloromethane eliminates the printing lines with a greater time of exposure of the component to it, this being a factor dependent only on the height of the layer.
- The chemical treatment with dichloromethane can get into the interior of the component if it is not manufactured anticipating its subsequent treatment, mainly due to a longer exposure time or an excessive layer height, as well as an insufficient wall thickness.
- It has been possible to determine a series of groups with printing variables (layer height, wall thickness and filling) in combination and exposure times to chemical treatment suitable for their use.
- Scanning electron microscopy has reflected a smooth surface without any kind of defect or discontinuity, providing higher magnifications with an excellent microscopic finish and higher quality than the untreated component.
- FTIR spectra reflect the non-existence of material degradation after chemical surface treatment, even after the longest exposure times.

Based on the above and with the partial conclusions realized, it can be concluded that chemical surface treatment with dichloromethane is an excellent option, as it is fast, economical and provides a very good surface finish for the treatment of 3D printing by FDM technology of polycarbonate components. However, care must be taken with the printing variables as well as the exposure times to ensure the treatment eliminates the printing lines and does not affect the structure of the component.

In short, the usefulness of this surface treatment for the elimination of printing lines has been demonstrated. At the same time, printing variables and exposure times to the treatment that create a quality surface on the samples, without deterioration of the same, have been provided. These variables have been presented in a graphic scheme, in which various viable options can quickly be chosen to carry out the treatment successfully. This study can therefore be extrapolated to professional processes with various variables and in an infinite number of cases.

## Figures and Tables

**Table 1:** Polycarbonate properties.

Mechanical Properties				
	Injection Molding		3D Printing	
	Typical Value	Test Method	Typical Value	Test Method
Tensile modulus	-	-	2134 MPa	ISO 527
Tensile stress at break	-	-	76.4 MPa	ISO 527
Elongation at break	-	-	6.4 %	ISO 527
Flexural strength	-	-	111 MPa	ISO 178
Flexural modulus	-	-	2.4 MPa	ISO 178
Izod impact strength (23 °C)	-	-	4.1 KJ/m^2^	ISO 180
Hardness (Shore D)	-	-	82	ISO 7619
**Electrical Properties**				
Dissipation factor (1 MHz)	-	-	0.005	ASTM D150-11
Dielectric constant (1 MHz)	-	-	2.62	ASTM D150-11
**Thermal Properties**				
Melt mass-flow rate	32–35 g/10 min	300 °C, 1.2 kg		
Glass transition	112–113 °C	DSC, 10 °C/min		
**Other Properties**				
Specific gravity	1180–1200 Kg/m^3^		ASTM D792	

**Table 2:** Characteristics of Dichloromethane used as a chemical treatment.

Chemical Formula	Molar Mass (g/mol)	Boiling Point (°C)	Density at 20 °C (kg/m^3^)
CH_2_Cl_2_	84.93	39.75	1320

**Table 3:** Groups of samples manufactured with different printing variables.

Wall Thickness	Layer Height	Infill Density
0.6 mm	0.06 mm	20%
	0.18 mm	20%
	0.30 mm	20%
1 mm	0.06 mm	20%
	0.18 mm	20%
	0.30 mm	20%
1.4 mm	0.06 mm	20%
	0.18 mm	20%
	0.30 mm	20%
1.8 mm	0.06 mm	20%
	0.18 mm	20%
	0.30 mm	20%
Density 100%	0.06 mm	100%
	0.18 mm	100%
	0.30 mm	100%

**Table 4:** Dimensional variation for each exposure time and layer height of 0.06 mm.

Layer Height of 0.06 mm	
Exposure Times	Dimensional Variation (mm)
1	0.03 ± 0.00
2.5	0.05 ± 0.00
5	0.06 ± 0.00
7.5	0.07 ± 0.00
10	0.08 ± 0.00

**Table 5:** Dimensional variation for each exposure time and layer height of 0.18 mm.

Layer Height of 0.18 mm	
Exposure Times	Dimensional Variation (mm)
1	0.04 ± 0.00
2.5	0.10 ± 0.00
5	0.18 ± 0.00
7.5	0.20 ± 0.00
10	0.23 ± 0.01

**Table 6:** Dimensional variation for each exposure time and layer height of 0.30 mm.

Layer Height of 0.30 mm	
Exposure Times	Dimensional Variation (mm)
1	0.05 ± 0.00
2.5	0.09 ± 0.00
5	0.17 ± 0.00
7.5	0.30 ± 0.01
10	0.35 ± 0.01

**Table 7:** Simple compression strength test for different wall thicknesses and exposure times with a layer height of 0.06 mm.

		Wall Thickness				
		0.6 mm	1 mm	1.4 mm	1.8 mm	Full Infill
**Exposure Times**	1 s.	OK	OK	OK	OK	OK
	2.5 s.	OK	OK	OK	OK	OK
	5 s.	OK	OK	OK	OK	OK
	7.5 s.	NULL	OK	OK	OK	OK
	10 s.	NULL	NULL	OK	OK	OK

**Table 8:** Simple compression strength test for different wall thicknesses and exposure times with a layer height of 0.18 mm.

		Wall Thickness				
		0.6 mm	1 mm	1.4 mm	1.8 mm	Full Infill
**Exposure Times**	1 s.	OK	OK	OK	OK	OK
	2.5 s.	OK	OK	OK	OK	OK
	5 s.	NULL	OK	OK	OK	OK
	7.5 s.	NULL	NULL	OK	OK	OK
	10 s.	NULL	NULL	NULL	OK	OK

**Table 9:** Simple compression strength test for different wall thicknesses and exposure times with a layer height of 0.30 mm.

		Wall Thickness				
		0.6 mm	1 mm	1.4 mm	1.8 mm	Full Infill
**Exposure Times**	1 s.	NULL	NULL	OK	OK	OK
	2.5 s.	NULL	NULL	NULL	OK	OK
	5 s.	NULL	NULL	NULL	OK	OK
	7.5 s.	NULL	NULL	NULL	NULL	OK
	10 s.	NULL	NULL	NULL	NULL	OK

**Table 10:** Groups of samples with different printing variables and exposure times suitable for chemical surface treatment.

		**Wall Thickness and Layer Height of 0.06 mm**				
		**0.6 mm**	**1 mm**	**1.4 mm**	**1.8 mm**	**Full Infill**
	1 s.	NULL	NULL	NULL	NULL	NULL
**Exposure Times**	2.5 s.	OK	OK	OK	OK	OK
	5 s.	OK	OK	OK	OK	OK
	7.5 s.	NULL	OK	OK	OK	OK
	10 s.	NULL	NULL	OK	OK	OK
		**Wall Thickness and Layer Height of 0.18 mm**				
		**0.6 mm**	**1 mm**	**1.4 mm**	**1.8 mm**	**Full infill**
	1 s.	NULL	NULL	NULL	NULL	NULL
**Exposure Times**	2.5 s.	NULL	NULL	NULL	NULL	NULL
	5 s.	NULL	OK	OK	OK	OK
	7.5 s.	NULL	NULL	OK	OK	OK
	10 s.	NULL	NULL	NULL	OK	OK
		**Wall Thickness and Layer Height of 0.30 mm**				
		**0.6 mm**	**1 mm**	**1.4 mm**	**1.8 mm**	**Full infill**
	1 s.	NULL	NULL	NULL	NULL	NULL
**Exposure Times**	2.5 s.	NULL	NULL	NULL	NULL	NULL
	5 s.	NULL	NULL	NULL	NULL	NULL
	7.5 s.	NULL	NULL	NULL	NULL	OK
	10 s.	NULL	NULL	NULL	NULL	OK
